# Abnormal Adrenal Mass Presents as Proximal Epithelioid Sarcoma

**DOI:** 10.1155/2020/8864218

**Published:** 2020-09-22

**Authors:** Valeria Pereira Martinez, Marilin Nicholson, Trushar Patel

**Affiliations:** USF Health Morsani College of Medicine, 560 Channelside Drive, Tampa, FL 33602, USA

## Abstract

Epithelioid sarcoma (ES) is a rare malignant mesenchymal neoplasm that accounts for less than one percent of all soft-tissue sarcomas. Only two cases of ES involving the adrenal gland were found after a literature review. We report a case of an 82-year-old female initially presenting with right flank pain who was subsequently found to have an incidental left adrenal mass on CT imaging. After appropriate diagnostic workup, the patient underwent surgical resection. A diagnosis of ES was made from the histopathological analysis. The characteristic findings of ES are epithelioid cells with rhabdoid morphology and moderate eosinophilic cytoplasm. Immunohistochemical findings are significant for positive staining for epithelial markers, cytokeratins, vimentin, and CD34, and loss of INI-1 stain. Due to the aggressive nature and limited data of ESs, the standard treatment continues to remain wide surgical excision.

## 1. Introduction

Epithelioid sarcoma (ES) is a malignant mesenchymal neoplasm that shows some level of epithelial differentiation [1–4]. It is a very rare sarcoma that accounts for less than one percent of all soft tissue sarcomas [1, 2]. There are two subtypes: classic and proximal [1–4]. The classic subtype presents more commonly in adolescents and young adults in distal extremities, whereas the proximal subtype tends to present in older adults in proximal soft tissue [1–4]. The cytomorphology of the proximal type tends to be higher grade than the classic type, and some of its malignant cells have a rhabdoid phenotype [1–4]. We report a case of proximal ES that is presented as an adrenal mass. This is the third reported ES presenting as an adrenal mass. The first reported primary adrenal ES was in 2017 by Alikhan et al. [3] and the second was in 2019 by Huang et al. [4].

## 2. Case Report

**Figure 1:**
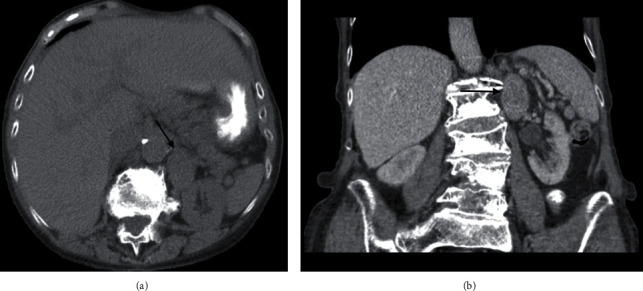
Initial CT abdomen showed a 3.3 cm left adrenal indeterminate mass.

**Figure 2:**
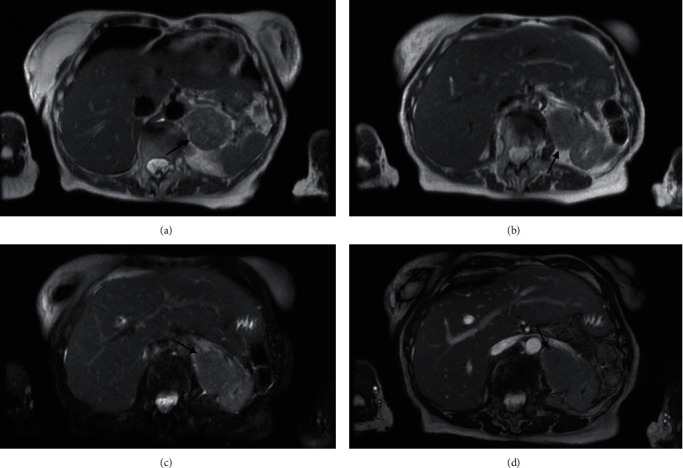
Follow-up MRI, ten months later, showed significant enlargement of the left adrenal mass, measuring 6.5 × 5.1 × 5.0 cm.

An 82-year-old female initially presented with right flank pain. The patient's past medical history was noncontributory. Family history was significant for breast, stomach, uterine, and colon cancer. The initial CT abdomen with and without contrast showed a 3.3 cm left adrenal indeterminate mass with an absolute and relative washout of 21.9% and 10.1%, respectively, which did not meet the washout criteria for an adrenal adenoma (Figure 1). Metabolic workup with a comprehensive metabolic panel, plasma metanephrines, and dexamethasone suppression test was all normal. The patient showed no signs of refractory hypertension or stigmata of Cushing's disease. Based on the negative metabolic workup and relatively small size of the adrenal mass, the patient was placed on active surveillance. A follow-up MRI, ten months later, showed significant enlargement of the left adrenal mass, which at the time measured 6.5 × 5.1 × 5.0 cm (Figure 2). Repeat metabolic testing was again negative. Surgical treatment was recommended, and the patient underwent left open adrenalectomy. Laparoscopic/robotic adrenalectomy was not considered because of the suspicion of adrenal cortical carcinoma and the data that has been presented regarding the increased risk of peritoneal dissemination with laparoscopic surgery in these cases [5]. The intraoperative findings included limited access to the mass due to the proximity of the spleen and the mass being significantly involved with the renal hilum. To aid in access to the mass, a splenectomy had to be performed. The adrenal mass was densely adherent to the renal hilum, specifically the renal vein. After unsuccessful attempts to safely release the renal hilum, the decision was made to perform a nephrectomy, with the adrenalectomy and mass excision. The postoperative course was complicated by congestive heart failure exacerbation which was ultimately managed with diuretics. The remainder of the postoperative course was uneventful, and the patient was ultimately discharged on postoperative day 5. At four months post-op, the patient is doing well with a creatinine of 1.62. Follow-up imaging has been delayed due to the COVID-19 pandemic.

**Figure 3:**
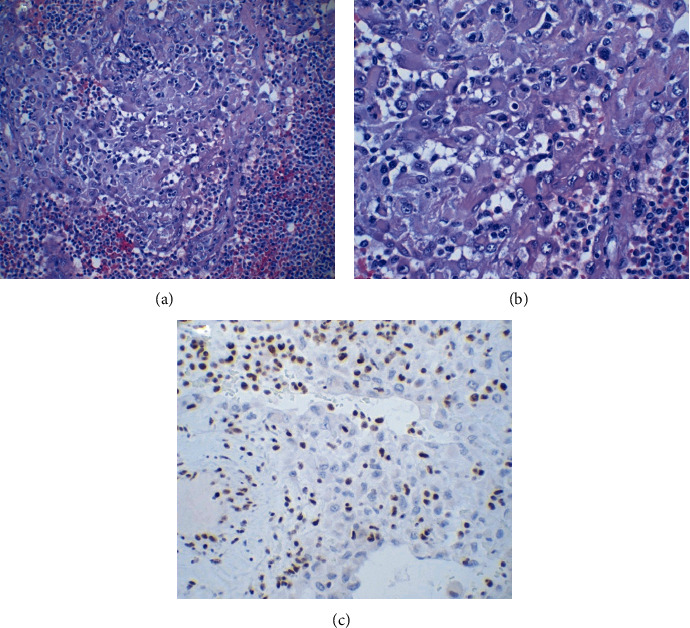
Immunohistochemical findings postresection. (a) Malignant epithelioid cells with moderate eosinophilic cytoplasm and rhabdoid features along with prominent lymphoid infiltrate (200x). (b) Higher magnification (400x). (c) Loss of INI-1 stain in neoplastic cells; background inflammatory cells stained positive with INI-1.

The pathologic analysis of the spleen found no diagnostic abnormalities, and the analysis of the left kidney was only significant for benign cysts. Macroscopic analysis of the resected specimen showed a well-defined, 7 cm tumor mass which was actually adjacent to the adrenal gland. One lymph node was analyzed and showed no malignancy. The microscopic analysis of the tumor showed malignant epithelioid cells with moderate eosinophilic cytoplasm and rhabdoid features (Figure 3). Prominent lymphoid infiltrate was associated with the tumor cells (Figure 3). Areas of tumor necrosis were also identified. The tumor cells stained positive for keratin Cam 5.2 and CD34, and they showed negative staining for INI-1.  Figure 3(c) demonstrates the loss of INI-1 stain in neoplastic cells; background inflammatory cells stained positive with INI-1. Focal immunoreactivity for calretinin was identified. Additionally, the tumor cells stained negative for smooth muscle actin, desmin, myoD1, myogenin, S100, inhibin, synaptophysin, CD31, ERG, and D240. The diagnosis of ES proximal type was made.

## 3. Discussion

ESs are very rare neoplasms that tend to have an aggressive clinical course and tend to recur [1–4]. The size of ES neoplasms can get quite large with a median diameter of 4 cm, and they are typically painless [1–4]. In this case, the ES neoplasm presenting as an adrenal mass was an incidental finding and was quite large as well—measuring 6.5 × 5.1 × 5.0 cm. Differential diagnoses included adenoma or adrenocortical carcinoma. The diagnosis of ES is done with histopathology [2, 4]. ESs are composed of epithelioid cells with rhabdoid morphology and moderate eosinophilic cytoplasm, as was seen in our case [1–4]. The neoplastic cells in the proximal subtype tend to exhibit prominent nucleoli and vesicular nuclei [1–3]. Signs of chronic inflammation, such as the presence of lymphocytes, are also associated with ESs [1–3]. The common immunohistochemical findings are positive staining for epithelial markers, cytokeratins, vimentin, and CD34—which helps distinguish from carcinoma [1–4]. Other immunohistochemical findings are negative staining for CD31, nuclear INI1, and S100 [1–4]. In terms of prognosis, the reported probability of the recurrence of ES varies in the literature with a range from 34% to 87% and 30% to 50% for metastasis [1, 4]. The five-year survival rates reported also vary in the literature with a range of 49-75% depending on prognostic factors [1]. Factors that are associated with a worse prognosis for ESs are proximal and deep location, rhabdoid features, large size, older age, male sex, necrosis, and vascular invasion [1]. In our case, our patient has several of these negative prognostic factors: proximal and deep location, rhabdoid features, necrosis, large size, and older age, therefore yielding her a potential worse prognosis. The standard treatment for ESs that present without metastasis is wide surgical excision, and some patients receive adjuvant high-dose chemotherapy or radiotherapy to help prevent local relapse [1, 4]. Due to the tendency of relapse from ESs, follow-up after surgical excision is imperative. Our patient is currently 3 months from her surgical resection with no evidence of recurrence or metastatic disease on axial imaging. Based on the limited evidence of adjuvant therapy, we have continued her on active surveillance with repeat scans scheduled for 3 months post-op.

## 4. Conclusion

In summary, we report a case of an ES proximal type that is presented as an incidental adrenal mass. The diagnosis of ES is dependent on histopathology. The significant findings for ESs are epithelioid cells with rhabdoid morphology, moderate eosinophilic cytoplasm, positive staining for epithelial markers, cytokeratins, vimentin, and CD34, and loss of INI-1 stain in neoplastic cells. Due to the aggressive nature and limited data of ESs, the standard treatment continues to remain wide surgical excision.

## Data Availability

All data underlying the results are available as part of the article, and no additional source data are required.
